# Real-Time Lightweight Weld Seam Keypoint Detection and Tracking via an Improved SimCC with a Unified Three-Keypoint Formulation

**DOI:** 10.3390/s26092861

**Published:** 2026-05-03

**Authors:** Shenkuo Wang, Xiangjie Huang, Ang Gao, Chao Chen, Fuxin Du

**Affiliations:** 1 School of Mechanical Engineering, Shandong University, 17923 Jingshi Road, Jinan 250061, China; shenkuowang@mail.sdu.edu.cn (S.W.); 202414363@mail.sdu.edu.cn (A.G.); chaochen@sdjtu.edu.cn (C.C.); 2 Shantui Construction Machinery Co., Ltd., Jining 272073, China; huangxiangjie@shantui.com; 3 School of Rail Transportation, Shandong Jiaotong University, Jinan 250357, China

**Keywords:** weld seam, keypoint detection, SimCC, sub-pixel localization, log-domain quadratic peak refinement, lightweight network

## Abstract

Reliable weld seam perception remains challenging in industrial environments, where arc light, spatter, smoke, and varying seam geometries can seriously degrade visual sensing. These disturbances make it difficult to achieve a unified representation, accurate localization, and real-time inference at the same time. To address this problem, this paper presents an end-to-end lightweight framework for weld seam keypoint detection and tracking based on an improved SimCC. A unified three-keypoint formulation is introduced to represent different weld geometries by using one seam center point and two orientation reference points, thereby supporting a perception-to-control mapping in which position control and orientation control are decoupled. In addition, a lightweight C3k2-based backbone is designed, and a non-parametric log-domain quadratic peak-refinement decoder is proposed to alleviate the discretization-induced quantization error of SimCC classification distributions without adding model parameters. Experiments show that the proposed model contains only 1.4 M parameters, achieves 17.01 ms CPU inference latency, and obtains a detection accuracy of 1.89 px MAE. In curved weld seam tracking experiments with the integrated robotic system, it further achieves an average trajectory tracking error as low as 0.159 mm and an average orientation error of 3.738°, demonstrating its real-time accuracy and robustness for industrial welding applications.

## 1. Introduction

In high-end equipment manufacturing, welding is a fundamental process for joining steel structures, ship components, and key automotive parts [1,2]. Weld formation quality directly affects structural integrity and service life [3]. As intelligent manufacturing places increasing demands on flexibility and consistency, line-laser active structured-light vision has gradually replaced traditional teach-and-playback procedures. Owing to its non-contact measurement capability, rich geometric information, and stronger resistance to ambient-light interference, this technique has been widely used for weld-seam deviation correction and robotic guidance [4]. Although sensing hardware and optical components have become relatively mature, welding scenes are still affected by arc glare, spatter, smoke, surface reflections, and variations in seam geometry. Under real-time constraints, extracting weld features from images with both robustness and accuracy therefore remains a major barrier to engineering deployment.

Early studies used traditional digital image processing techniques (DIPT) [5,6,7] to extract laser center lines, relying on filtering, threshold segmentation and morphological operations, the process is relatively simple and can be explained; however, the thresholds adjusted by manual clues and experience are easily affected by changes in operating conditions and sudden noise fluctuations. In recent years, deep learning has made weld perception more adaptable to appearance variations. Existing methods mainly include object-detection models that localize the Region of Interest (ROI) to suppress background clutter [8,9], semantic-segmentation methods that densely label laser-stripe pixels [10,11], and Generative Adversarial Networks (GANs) for image enhancement and denoising [12,13]. Although these methods have improved weld perception, they usually output bounding boxes, masks, or enhanced images rather than the geometric points directly required for robot control.

In practical applications, these methods are often used in cascaded hybrid pipelines that combine deep models with traditional post-processing. A typical workflow first uses a network to generate bounding boxes, masks, or enhanced images, and then computes the final coordinates through conventional procedures such as geometric-center estimation or skeleton extraction [14,15]. Cheng and Jin [16] evaluated this type of hybrid pipeline for weld feature extraction. Their YOLOv8n+DIPT detection scheme achieved an RMSE of 11.489 px with a latency of 79.2 ms, while the Fast-SCNN+DIPT segmentation scheme reduced the RMSE to 4.786 px but still required 56.5 ms. These results suggest that improving the upstream detection or segmentation stage does not necessarily remove the accuracy and latency limits introduced by cascaded coordinate extraction. This indirect pipeline has several limitations. Error accumulation is difficult to avoid because uncertainty from deep predictions and errors from post-processing are combined, which increases the final localization error and makes deployment more complicated. The computation is also not well matched to the tracking task. When a network regresses many bounding-box parameters, performs pixel-wise classification over the whole image, or generates high-fidelity images, it learns a large amount of texture or region-level information. Weld tracking, however, usually requires only a small number of geometric keypoint coordinates, so part of the computation is spent on details that are only weakly related to the control objective [17]. Moreover, when the final coordinates are obtained through non-differentiable traditional operations, the coordinate error cannot be backpropagated through the whole pipeline. As a result, the model cannot be directly optimized for geometric localization accuracy.

To solve the problem, researchers used the idea of pose estimation, and also introduced a key point detection model. They restated the task as directly performing weld key point prediction, reducing the reliability problem of post-processing, and allowing the network to learn the geometric topology of welds. Heatmap-based regression is relatively common in existing weld key point methods. Zou et al. [12] combined a generative adversarial network (GAN) with a U-shaped architecture to generate probability heatmaps to suppress strong arc light interference. Even so, resolution recovery often relies on complex upsampling structures, which leads to large computational overhead, and the positioning accuracy is also limited by discrete grids. Deng et al. [18] proposed a real-time extraction method based on CenterNet. This method combines Gaussian heatmaps with an offset branch and a weld type classification branch. This method has 99.359% weld type classification accuracy, 1.754 pixel average extraction error and 32.186 millisecond average processing time on three typical welds.The results show that key point detection related to heat maps for weld feature extraction is effective. And its output structure is related to predefined weld categories, while the number of feature points varies with the weld type. An offset branch is also needed to calculate the downsampled discrete residuals. Mobaraki et al. [19] adopted stacked hourglass networks for key point localization. Multi-level cascading can improve robustness, but the computational burden is difficult to bear on low-power personal terminals, and the rough output resolution also limits the positioning accuracy. The heatmap paradigm has ongoing trade-offs among output resolution, structural complexity, and coordinate accuracy.

Beyond the trade-off between accuracy and speed, geometric differences among weld seams also limit algorithm generalization. Zou and Zeng [10] proposed a lightweight SOLOv2-based segmentation method combined with ECO tracking, achieving a CPU latency of 34.2 ms and a mean absolute welding error of about 0.20–0.21 mm with only 0.224 M parameters and 0.391 G FLOPs. However, the network still outputs masks rather than coordinates. As a result, the final seam feature point must be obtained through skeletonization and tracking, which prevents direct end-to-end optimization with respect to the control coordinates. Gao et al. [20] adopted a “case-by-case” strategy that divides welds into multiple categories for detection. Although this strategy can handle multi-shape recognition, overly fine category partitioning increases the annotation burden and disrupts the shared geometric relations among welds, thereby increasing the difficulty of classification prediction. Zhao et al. [21] proposed the DeepKP framework to improve keypoint extraction under multiple arc-light interference, reporting an average WSKP locating error of 1.75 px and an average seam tracking error of 0.336 mm. However, its core module still follows a “classify-first, regress-later” logic: it must explicitly recognize weld semantic categories to generate specific prior regions, and then extract coordinates within those regions. This strong coupling to class priors implies that a unified annotation and geometric representation system across weld types has not yet been established. The model generalization is therefore constrained by predefined training categories, and it is prone to failure for non-standard or hybrid weld geometries. Consequently, establishing a unified geometric representation that can be shared across weld types, and completing end-to-end detection and optimization within a single standardized model, remains an open problem.

Simple Coordinate Classification (SimCC) reformulates keypoint localization by replacing direct coordinate regression or dense two-dimensional (2D) heatmap estimation with two one-dimensional coordinate classification tasks [22]. This heatmap-free formulation is well suited to lightweight weld feature extraction because it avoids high-resolution heatmaps, produces sparse coordinate distributions for the required keypoints, and preserves fine-grained coordinate discrimination under limited computational resources. However, SimCC alone is not sufficient for robotic weld tracking. Heterogeneous seams require semantically consistent keypoints, the perception output must be easily converted into trajectory and torch-pose commands, and the detector must remain lightweight enough for CPU-side industrial deployment.

To meet these requirements, this paper proposes an end-to-end framework that combines a unified three-keypoint formulation with an improved lightweight SimCC detector. Each weld is represented by one seam center point for trajectory tracking and two orientation reference points for local cross-sectional pose estimation, forming a fixed-dimensional, control-oriented output across weld types. A compact C3k2-based backbone is used to reduce computational cost, while a non-parametric log-domain quadratic peak-refinement decoder refines coordinates from SimCC distributions without introducing trainable parameters.

The main contributions are summarized as follows:

(1) **Unified control-oriented three-keypoint formulation:** We introduce a fixed three-keypoint representation, composed of one seam center point and two orientation reference points, to describe heterogeneous weld seams with consistent semantics. By decoupling trajectory-position correction from orientation estimation, this formulation directly connects visual perception with robot control.
(2) **Lightweight SimCC with non-parametric coordinate refinement:** Using a lightweight C3k2-based backbone, we train SimCC with Gaussian soft labels and KL-divergence supervision, and propose a non-parametric log-domain quadratic peak-refinement decoder. This design enables sub-pixel coordinate decoding and reduces discretization-induced coordinate bias without increasing the parameter budget, while preserving an end-to-end pipeline suitable for real-time CPU-side deployment.
(3) **System-level validation for industrial closed-loop control:** We implement an integrated welding setup that couples visual perception with robot control, and conduct trajectory-tracking and orientation-adjustment tests on representative planar curved welds. The experiments verify that the proposed framework achieves a practical balance among model size, CPU latency, pixel-level localization accuracy, and closed-loop tracking performance.

## 2. Experimental Platform

**Figure 1:**
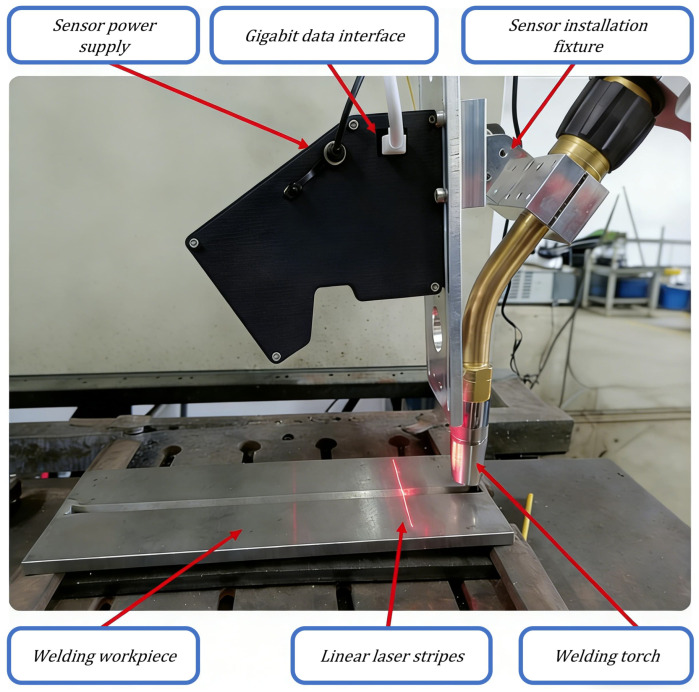
Line-laser structured-light sensor prototype used in the experiments.

To evaluate the proposed algorithm under practical operating conditions, we developed an integrated robotic welding vision system. The platform comprises a robotic subsystem, a vision sensing subsystem, a welding subsystem, and a central control computer. The corresponding hardware configuration is summarized in Table 1. The vision module employs a custom-developed structured-light sensor assembly, as shown in Figure 1. The sensor mainly includes a HIK board-level industrial camera, a 660 nm narrow-band filter, a laser projector that emits a single 660 nm laser line, and a control circuit for adjusting the laser intensity. The camera is equipped with a 25 mm industrial lens, and the acquired images used in this study have a resolution of 1280×1080 pixels. During image acquisition, the exposure setting and laser brightness were adjusted according to the welding conditions to obtain a clear laser stripe and reduce saturation caused by arc glare.

During welding, the structured-light sensor projects the laser line onto the workpiece surface, forming stripe information that represents the surface profile. The sensor simultaneously captures laser images, and the algorithm then computes the weld seam pixel coordinates in the 2D image coordinate system. Based on optical triangulation, the detected 2D keypoints are first converted into 3D points in the sensor coordinate system using the calibrated camera parameters and structured-light plane. Then, with the hand–eye calibration result and the robot pose, the weld seam position is transformed into the robot base frame for trajectory tracking and pose adjustment [23]. In this study, camera calibration was performed using checkerboard images, the structured-light plane was obtained by fitting the extracted laser centerline points, and the hand–eye transformation was calibrated before the welding experiments.

The platform implements the complete execution chain for closed-loop tracking, covering image acquisition, visual inference, coordinate transformation, controller communication, and robot motion updating. The system was tested under practical welding conditions, where arc glare, spatter, smoke, surface reflection, and local laser-stripe occlusion may interfere with structured-light imaging.

## 3. Unified Three-Keypoint Formulation and Dataset Construction

### 3.1. Unified Three-Keypoint Formulation

To accommodate the morphological diversity of heterogeneous weld seams in industrial practice, support unified modeling across different weld types, and meet the real-time control requirements of robotic systems, we propose a unified three-keypoint formulation with a standardized annotation scheme. Inspired by the work of Yu et al. [24], the weld seam geometry is abstracted into three keypoints: one seam center point (*P_c_*) and two orientation reference points (*P_l_* and *P_r_*).

*P_c_* denotes the seam center point and serves as the target point followed by the robot Tool Center Point (TCP). *P_l_* and *P_r_* are typically placed on the two sides of the weld profile; together with *P_c_*, they provide local orientation cues for the cross-section. The geometric relationship among *P_l_*, *P_c_*, and *P_r_* is used to compute the normal information of the weld cross-section, thereby providing a stable basis for welding torch pose estimation. With this definition, weld perception is formulated at the network level as a sparse keypoint localization problem with a fixed output size, while the resulting standardized output can be directly used in a perception-to-control mapping for position and orientation control.

This formulation differs from existing weld-feature representations in its fixed semantics and control orientation. Instead of using weld-category-dependent feature points, bounding boxes, segmentation masks, or hand-crafted geometric rules, the proposed formulation assigns the same physical meaning to a fixed-dimensional keypoint triplet across weld types. Thus, the perception output becomes a compact geometric state that can be directly mapped to trajectory correction and torch-pose estimation, rather than an intermediate visual description requiring additional post-processing.

**Figure 2:**
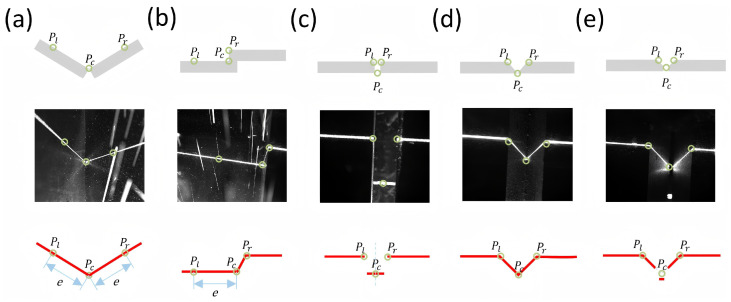
Unified three-keypoint annotation scheme for representative weld types: (**a**) Fillet weld; (**b**) Lap weld; (**c**) Butt-I weld; (**d**) Butt-V weld; (**e**) Butt-Y weld.

Based on the above definition, we formulate standardized three-point annotation rules for five typical joint types (see Figure 2):

In this formulation, *P_c_* is referred to as the *seam center point*, and *P_l_*/*P_r_* are referred to as the *left/right orientation reference points*.

- **Fillet Weld:** *P_c_* is defined as the root vertex of the groove. *P_l_* and *P_r_* are located at a preset pixel distance *e* (set to 300 pixels in this study) extending from *P_c_* along both sides of the laser stripe profile.
- **Lap Weld:** *P_c_* is defined as the inner corner of the step, and *P_l_* is the outer corner. *P_r_* is obtained by extending the same distance *e* from *P_c_* along the laser stripe on the opposite side.
- **Butt-I Weld:** *P_l_* and *P_r_* are defined as the two endpoints on the upper surface of the weld seam. *P_c_* is the midpoint of the line connecting the two bottom endpoints of the weld seam.
- **Butt-V Weld:** *P_c_* is defined as the valley vertex of the “V” shape. *P_l_* and *P_r_* are the two intersections between the upper edge of the groove and the laser stripe.
- **Butt-Y Weld:** *P_l_* and *P_r_* are defined as the two outermost endpoints on the upper edge of the groove. *P_c_* is the intersection of the extended lines from the two sloped sections in the middle area of the groove, representing the theoretical “V-shaped” root.

The preset distance *e* used for the Fillet and Lap welds is an empirical task-related hyperparameter rather than an arbitrary constant. When *e* is too small, the two orientation reference points lie close to *P_c_*, making the estimated local orientation more susceptible to laser-stripe noise, arc-induced halo, or minor annotation deviations. If *e* is too large, the reference points may fall outside the stable local weld structure, where they can be affected by irrelevant surface variations or partial stripe occlusion. Under the current camera field of view, image resolution, and typical weld scale, e=300 pixels provides a sufficiently long and stable orientation baseline while keeping the reference points within the local weld profile in most samples. For different sensors, fields of view, or weld sizes, this parameter should be re-tuned according to the corresponding imaging scale.

Compared with conventional approaches, this unified feature representation reduces the recognition of multiple weld types to a single sparse keypoint localization task with a fixed output dimension. As a result, one deep learning model can handle all weld types without redesigning the model structure for each heterogeneous geometry, which improves universality and generalization. The control requirements are reflected in the definitions of *P_c_*, *P_l_*, and *P_r_*: *P_c_* is used as the trajectory-tracking reference to guide TCP position correction, whereas *P_l_* and *P_r_* constrain the local cross-sectional direction and provide explicit cues for estimating the torch normal and the feeding pose. Since the keypoint semantics are anchored in joint structures rather than merely in geometric corners, the proposed annotation is easier to interpret and tends to yield more consistent labels. At the same time, the network is encouraged to focus on control-relevant geometric semantics, instead of relying on redundant appearance variations.

### 3.2. Dataset Construction

Following the unified three-keypoint formulation, we carried out purpose-oriented data collection in a real industrial welding setting. The recorded scenes include common welding disturbances, such as intense arc light, spatter, smoke, surface reflection, and local laser-stripe occlusion, and cover the five joint types described above. In total, 6671 grayscale images at a resolution of 1280×1080 pixels were acquired.

Keypoint annotation strictly followed the unified three-keypoint rules defined in Section 3.1 and was completed frame by frame with pixel-level precision using the open-source tool LabelMe. Before annotation, the keypoint definitions and the ordering of *P_l_*, *P_c_*, and *P_r_* were standardized to reduce operator-dependent ambiguity. For keypoints partially occluded by arc light, spatter, smoke, or local stripe saturation, annotators inferred their positions according to laser-stripe continuity, local geometric context, and visible structural edges. For Fillet and Lap welds, where the orientation reference points are defined by the preset distance *e*, the reference direction was estimated from the visible stripe branch when the nominal point was locally occluded, and the point was then determined by extending from *P_c_* along that branch by *e*. After annotation, all labels were checked for consistency with the predefined rules. This strategy helps preserve the complete weld geometry under harsh imaging conditions, although a small amount of annotation uncertainty may remain in severely occluded cases.

After quality control, the 6671 images were randomly divided into a training set of 5364 images and a validation set of 1307 images at an 8:2 ratio. The per-type distribution is reported in Table 2: Fillet (train 1039, val 251), Lap (train 1001, val 251), Butt-I (train 1140, val 274), Butt-V (train 1172, val 282), and Butt-Y (train 1012, val 249). During training, online data augmentation was employed to enhance robustness against practical industrial disturbances, including varying seam orientations and rotating workpieces.

## 4. Lightweight Weld Seam Detection Network Based on Improved SimCC

To satisfy the real-time response and localization accuracy required in robotic welding, we design an end-to-end keypoint detection pipeline based on an improved SimCC framework. Unlike conventional two-stage methods, the proposed pipeline removes the post-processing steps that are typically applied after visual prediction. It combines temporally guided adaptive ROI cropping, a compact feature-extraction backbone, and a non-parametric peak-refinement decoder, enabling efficient weld-feature localization while maintaining high coordinate precision.

### 4.1. Overall Network Structure and Temporal Keypoint-Guided Adaptive ROI Cropping

**Figure 3:**
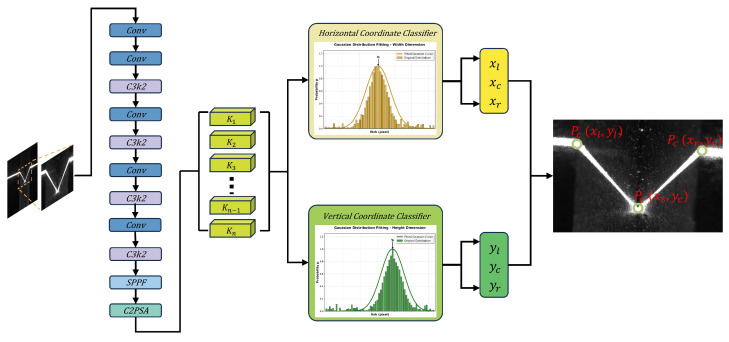
Overview of the proposed pipeline: ROI cropping, lightweight backbone, SimCC prediction, and non-parametric log-domain quadratic peak-refinement decoding.

As shown in Figure 3, the network performs end-to-end keypoint inference through full-frame initialization, ROI-based tracking, and sub-pixel decoding.

**Figure 4:**
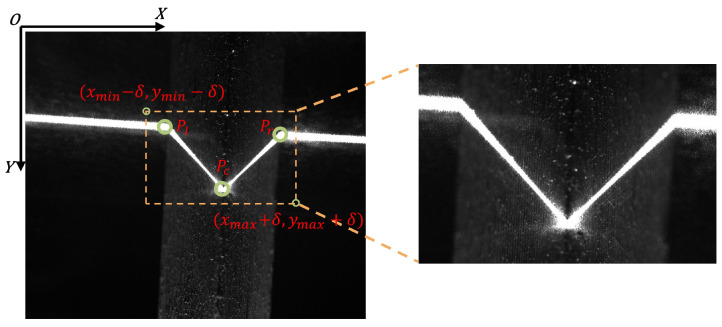
Schematic of temporally guided adaptive ROI cropping based on previous-frame keypoints.

To reduce interference from complex industrial backgrounds and lower the computational cost, the network uses a temporally guided adaptive ROI cropping scheme based on the detected keypoints. The weld keypoints consist of the seam center point *P_c_*, the left orientation reference point *P_l_*, and the right orientation reference point *P_r_*. The specific rules for ROI extraction are as follows:

- **Initial Frame Processing (Full-frame initialization):** For the first frame of the welding sequence, due to the lack of prior positional information, the system inputs the full-frame image into the network for global detection to obtain the initial keypoint coordinates.
- **Subsequent Frame Tracking (Temporal tracking):** During the welding process, leveraging the spatial continuity of the weld seam, the ROI bounding box is calculated based on the three keypoint coordinates $K_{t-1}={\left\{P_{c},P_{l},P_{r}\right\}}_{t-1}$ detected in the previous frame (t−1):
  (1) $$\begin{array}{cc} x_{\min} & =\max\;\left(0,\;\min(x_{c}^{t-1},x_{l}^{t-1},x_{r}^{t-1})-\delta \right),\\ x_{\max} & =\min\;\left(W_{0}-1,\;\max\left(x_{c}^{t-1},x_{l}^{t-1},x_{r}^{t-1}\right)+\delta \right) \end{array}$$
  (2) $$\begin{array}{cc} y_{\min} & =\max\;\left(0,\;\min(y_{c}^{t-1},y_{l}^{t-1},y_{r}^{t-1})-\delta \right),\\ y_{\max} & =\min\;\left(H_{0}-1,\;\max\left(y_{c}^{t-1},y_{l}^{t-1},y_{r}^{t-1}\right)+\delta \right) \end{array}$$

where δ is a preset margin (set to 80 pixels in this study), and $W_{0}$, $H_{0}$ denote the original image width and height. All coordinates in $K_{t-1}$ are defined in the original image coordinate system. The cropped ROI region has a width of $w_{\mathrm{roi}}=x_{\max}-x_{\min}+1$ and a height of $h_{\mathrm{roi}}=y_{\max}-y_{\min}+1$, which is subsequently resized to the fixed network input size $W_{\mathrm{in}}\times H_{\mathrm{in}}$ (set to 256×256 in this study) before being fed into the feature extraction network. The boundary clamping keeps the cropped ROI within the valid image extent. The ROI cropping procedure is shown in Figure 4.

The temporal ROI strategy uses the spatial continuity of robotic welding sequences, in which the inter-frame seam displacement is limited by the welding speed, camera frame rate, and robot motion constraints. Thus, the keypoints detected in the previous frame usually provide a reliable prior for locating the weld region in the current frame. The margin pixels adds contextual information around the three keypoints, tolerates moderate localization errors from the preceding frame, and reduces the risk of excluding the laser stripe or weld structure from the crop. However, the strategy may become unreliable when the previous frame is severely misdetected, when transient spatter or arc glare is strong, when the seam geometry changes abruptly, or when the target lies close to the image boundary. In practical deployment, the ROI can be enlarged, or full-frame re-initialization can be triggered, once abnormal geometry, low confidence, or boundary-near predictions are observed. Long-sequence drift and failure-recovery behavior will be further quantified in future work.

The cropped ROI image is fed into the feature extraction network to generate deep feature maps. Subsequently, the SimCC classification head decouples these features into 1D coordinate classification distributions for the horizontal (*X*) and vertical (*Y*) axes. Finally, log-domain quadratic peak-refinement decoding is applied to recover sub-pixel coordinates from the classification distributions and map them back to the original image coordinate system. After inference on the resized ROI, the predicted coordinates $\left({\hat{x}}_{\mathrm{in}},{\hat{y}}_{\mathrm{in}}\right)$ in the network input coordinate system are mapped back to the original image by applying the inverse scaling and the ROI origin offset:

(3) $$\hat{x}=x_{\min}+{\hat{x}}_{\mathrm{in}}\cdot \frac{w_{\mathrm{roi}}}{W_{\mathrm{in}}},\;\hat{y}=y_{\min}+{\hat{y}}_{\mathrm{in}}\cdot \frac{h_{\mathrm{roi}}}{H_{\mathrm{in}}}$$

where $\left({\hat{x}}_{\mathrm{in}},{\hat{y}}_{\mathrm{in}}\right)$ are the decoded keypoint coordinates in the $W_{\mathrm{in}}\times H_{\mathrm{in}}$ input space, $w_{\mathrm{roi}}=x_{\max}-x_{\min}+1$ and $h_{\mathrm{roi}}=y_{\max}-y_{\min}+1$ are the pixel dimensions of the cropped ROI in the original image, and $W_{\mathrm{in}},H_{\mathrm{in}}$ are the fixed network input dimensions.

### 4.2. Lightweight Feature Extraction Backbone Based on C3k2 Modules

**Figure 5:**
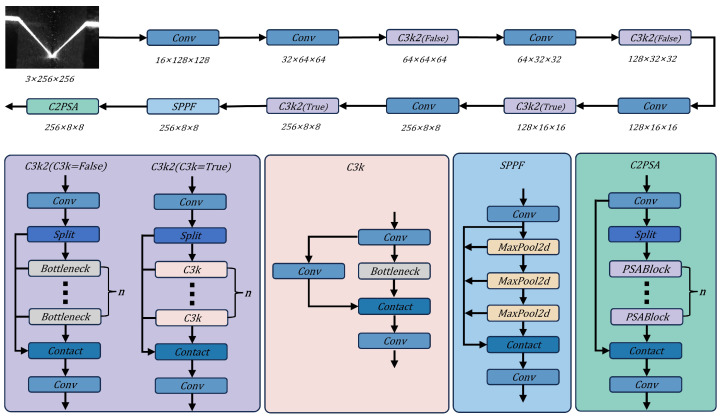
Architecture of the lightweight feature extraction backbone.

In the feature extraction stage, High-Resolution Networks (HRNet [25]) are often used in original SimCC implementations for their ability to preserve spatial geometry through parallel multi-resolution sub-streams. However, their complex cross-layer connections and large parameter count result in excessive computational costs, making them unsuitable for the millisecond-level real-time inference required by industrial embedded devices. Similar efficiency-oriented designs have also been emphasized in other real-time applied vision tasks, where lightweight feature extraction and attention mechanisms are used to preserve structural details while reducing computational cost [26]. To significantly improve inference speed while maintaining accuracy, this paper does not directly employ the complete YOLOv11 detector. Instead, it borrows the lightweight backbone design philosophy and several efficient feature-extraction components from YOLOv11 [27], including C3k2, SPPF, and C2PSA, while removing the object-detection head, bounding-box regression branch, objectness prediction, and other detection-specific structures. The resulting network is therefore redesigned as a compact feature extractor followed by SimCC coordinate-classification heads for sparse weld keypoint localization. The detailed backbone architecture is depicted in Figure 5.

The backbone is constructed using C3k2 (Cross Stage Partial (CSP) 3-convolution with K2 enhancement) modules and the C2PSA (C2 Position-Sensitive Attention) mechanism, which jointly form the feature extraction path. The C3k2 module can be regarded as a refined version of the preceding C2f structure. Within the CSP bottleneck, more compact convolutional operations and configurable kernel sizes are introduced to perform “split–merge” feature-map processing. This arrangement smooths gradient propagation and, meanwhile, suppresses redundant gradient responses while reducing the model’s floating-point operations (FLOPs).

SPPF (Spatial Pyramid Pooling - Fast) and C2PSA are further placed at the end of the deep feature extractor. With position-aware attention, C2PSA emphasizes responses in key weld regions, such as groove edges, laser-stripe intersections, and seam-center regions, without adding substantial computation, thereby weakening background interference caused by strong arc light and spatter.

Compared with HRNet, the C3k2-stacked lightweight backbone provides considerably higher computational efficiency. HRNet preserves high-resolution representations through parallel branches, whereas the proposed design relies on efficient downsampling, deeper semantic abstraction, and attention mechanisms to compensate for the loss of spatial detail. This structure is suitable for weld keypoint detection because the task mainly depends on sparse local geometric structures, such as groove edges, stripe intersections, and seam-center cues, rather than dense region-level prediction. The lightweight backbone encodes multi-scale texture and geometric semantics for heterogeneous welds, while the attention mechanism helps suppress disturbances caused by arc glare, spatter, and reflections. Experimental results indicate that this structure retains multi-scale feature extraction capability for heterogeneous welds, including extremely narrow butt seams and wide V-grooves, while reducing model weights by several-fold. The smaller model footprint improves memory efficiency and increases the frame rate, providing a reliable computational basis for high-frequency real-time tracking in complex welding environments.

### 4.3. SimCC Coordinate-Classification Head and Log-Domain Peak-Refinement Decoding

SimCC [22] formulates keypoint localization as two one-dimensional coordinate classification tasks along the horizontal and vertical axes, rather than using direct coordinate regression or dense two-dimensional heatmap estimation. This formulation is well matched to the proposed weld perception task, where only three sparse geometric keypoints need to be predicted. Compared with dense heatmap prediction, SimCC avoids high-resolution 2D response maps and is therefore more efficient for low-resolution inputs and CPU-side inference. It also preserves fine-grained coordinate discrimination, which is important for robotic welding because small pixel deviations may lead to measurable trajectory errors. Based on this property, we introduce an improved decoding strategy to reduce discretization-induced quantization errors.

#### 4.3.1. Coordinate-Classification Formulation

Many existing keypoint localization methods rely on 2D heatmap-based representations, such as FasterPose [28] and HRNet [25]. They typically generate a 2D Gaussian response centered at the ground-truth coordinate. To improve accuracy, upsampled heatmaps are often required [29], which increases computational cost. In addition, when the heatmap resolution differs from the original image resolution, quantization errors may occur.

In contrast, SimCC decouples localization into two independent classification tasks along the horizontal and vertical axes. By replacing 2D heatmap regression with dense one-dimensional bins, it provides fine-grained coordinate discrimination with a more compact output representation.

Specifically, SimCC first extracts keypoint feature representations via the backbone network. The subsequent classification head discretizes the continuous coordinate $x_{\mathrm{in}}\in \left[0,\;W_{\mathrm{in}}-1\right]$ in the resized network input space into classification labels $c_{x}\in \left\{0,1,\ldots ,W_{\mathrm{in}}\cdot k-1\right\}$. Here, *k* is the split factor, which controls the number of classification bins assigned to each input pixel. In all experiments, k=2, meaning that each pixel is subdivided into two coordinate bins. For an input of size $W_{\mathrm{in}}\times H_{\mathrm{in}}$, SimCC uniformly divides each axis into $W_{\mathrm{in}}\cdot k$ or $H_{\mathrm{in}}\cdot k$ bins, respectively:

(4) $${\mathrm{bins}}_{x}=\left\{0,1,\ldots ,W_{\mathrm{in}}\cdot k-1\right\},\;{\mathrm{bins}}_{y}=\left\{0,1,\ldots ,H_{\mathrm{in}}\cdot k-1\right\}$$

where k≥1. This means each input pixel is further subdivided into *k* sub-pixel grids. The model predicts the probability vectors $P_{x}$ and $P_{y}$ for the horizontal and vertical directions, respectively, using two simple linear layers.

#### 4.3.2. Non-Parametric Log-Domain Quadratic Peak-Refinement Decoding

**Figure 6:**
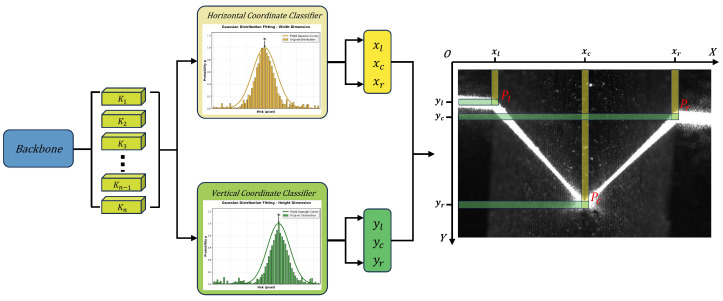
SimCCcoordinate classification and the proposed non-parametric log-domain quadratic peak-refinement decoding.

Although SimCC reduces the learning difficulty by converting coordinate regression into classification, its localization accuracy is still affected by the granularity of the discretized bins. To reduce this quantization error, we propose a non-parametric decoding strategy that is aligned with the training objective. The SimCC formulation and the proposed log-domain refinement are illustrated in Figure 6.

- **Label Smoothing and Distribution Prior**
  During training data preprocessing, we adopt the SA-SimDR (Soft-Argmax SimDR) strategy to reduce the overfitting risk of one-hot hard labels and preserve the spatial semantics of adjacent grids. For the *x*-axis, let $x_{\mathrm{gt}}$ denote the ground-truth coordinate in the resized network input coordinate system, where the ROI has been resized to $W_{\mathrm{in}}\times H_{\mathrm{in}}$. Thus, $x_{\mathrm{gt}}\in \left[0,\;W_{\mathrm{in}}-1\right]$. The *y*-axis is handled in the same way. The target probability distribution $T_{x}\left(n\right)$ is defined as a normalized discrete Gaussian centered at $x_{\mathrm{gt}}$, as given in Equation (5):
  (5) $${\tilde{T}}_{x}\left(n\right)=\exp\;\left(-\frac{{\left(n-x_{\mathrm{gt}}\cdot k\right)}^{2}}{2\sigma^{2}}\right),\;T_{x}\left(n\right)=\frac{{\tilde{T}}_{x}\left(n\right)}{\sum_{m=0}^{W_{\mathrm{in}}\cdot k-1}{\tilde{T}}_{x}\left(m\right)}$$
  where $W_{\mathrm{in}}$ is the network input width after ROI resizing, the *x*-axis classification length is $W_{\mathrm{in}}\cdot k$, $n\in \{0,1,\ldots ,W_{\mathrm{in}}\cdot k-1\}$ is the bin index, $x_{\mathrm{gt}}$ is the ground-truth coordinate in pixels, *k* is the split factor defined above, and σ is the standard deviation of the Gaussian soft label. In all experiments, k=2 and σ=4.0 are used; *k* controls the bin density along each axis, while σ controls the smoothness of the target probability distribution around the ground-truth coordinate. The explicit summation ensures that $T_{x}$ is a valid discrete probability distribution. This encourages the output distribution of the trained network to present a locally smooth peak around the target coordinate. However, standard Argmax decoding only selects the bin index $\hat{n}$ with the maximum probability and ignores the intra-bin sub-pixel information. Since each bin spans 1/k pixel, the worst-case quantization error of simple Argmax decoding is bounded by 1/(2k) pixel under an ideal noiseless setting.
- **Log-Domain Quadratic Peak Refinement**
  The proposed refinement does not require the predicted distribution to follow a strict Gaussian form over the entire axis. It only assumes that the neighborhood around the dominant peak is locally smooth and approximately unimodal under Gaussian soft-label supervision. Therefore, the decoder performs a local second-order approximation around the Argmax bin rather than fitting the whole distribution. When the distribution is multi-modal or severely corrupted by noise, the refinement gain may decrease; in such cases, the numerical protection described below makes the decoder fall back to a conservative Argmax-like estimate.
  Based on this local assumption, we propose a non-parametric log-domain quadratic peak-refinement decoding strategy to recover the continuous peak location near the maximum response.
  Under a local Gaussian approximation around the peak bin index $\hat{n}$ and its neighbors $\left\{\hat{n}-1,\hat{n},\hat{n}+1\right\}$, taking the natural logarithm of the Gaussian form yields Equation (6):
  (6) $$\ln\left(f\left(n\right)\right)=-\frac{{\left(n-\mu \right)}^{2}}{2\sigma^{2}}+\ln\left(\frac{1}{\sqrt{2\pi }\sigma }\right)$$
  Let $a=-\frac{1}{2\sigma^{2}}$; then the equation can be viewed as a quadratic function (parabola) with respect to the bin position *n*:
  (7) $$\ln f\left(n\right)=an^{2}+bn+d$$
  The true center μ of the Gaussian distribution corresponds to the vertex of this parabola, which, by the vertex formula, is $\mu =-\frac{b}{2a}$.
- **Implementation Details for Peak-Refinement Decoding**
  In the inference code, to reduce computation and concentrate on the most informative local neighborhood, we estimate the sub-pixel coordinate using only the peak bin and its immediate left and right neighbors. The steps are as follows:
  (1) **Peak Localization:** Find the integer index $\hat{n}\in \left\{0,1,\ldots ,W_{\mathrm{in}}\cdot k-1\right\}$ corresponding to the maximum probability via Argmax.
  (2) **Logarithmic Transformation:** Extract the probability values of the peak and its immediate neighbors, ${\hat{q}}_{\hat{n}-1},\;{\hat{q}}_{\hat{n}},\;{\hat{q}}_{\hat{n}+1}$, and convert them to the logarithmic domain:
    (8) $$L_{i}=\ln\left({\hat{q}}_{i}+\epsilon \right),\;i\in \left\{\hat{n}-1,\;\hat{n},\;\hat{n}+1\right\}$$
    where ϵ is a small constant (e.g., $10^{-6}$) used to prevent ln(0) and the associated numerical instability when ${\hat{q}}_{i}\approx 0$.
  (3) **Sub-pixel Offset Calculation:** A local parabola is fitted to these three points using finite differences, and the vertex offset Δx is then obtained. In the implementation, the offset is computed by Equation (9):
    (9) $$\Delta x=\frac{L_{\hat{n}-1}-L_{\hat{n}+1}}{2\;(L_{\hat{n}-1}-2L_{\hat{n}}+L_{\hat{n}+1})}$$
    The denominator is the discrete second derivative, which represents the local curvature of the fitted parabola, while the numerator corresponds to the first-derivative term. In practice, if $|L_{\hat{n}-1}-2L_{\hat{n}}+L_{\hat{n}+1}|<\tau$ with $\tau =10^{-4}$, the offset is clamped to zero. This safeguard prevents numerical instability when the local distribution is flat or abnormal, allowing the decoder to fall back to a conservative Argmax-like estimate in extreme cases rather than producing an unreliable large offset.
  (4) **Coordinate Recovery:** The final sub-pixel coordinate is recovered by Equation (10):
    (10) $${\hat{x}}_{\mathrm{final}}=x_{\min}+\frac{\hat{n}+\Delta x}{k}\cdot \frac{w_{\mathrm{roi}}}{W_{\mathrm{in}}}$$
    where $x_{\min}$ is the left boundary of the ROI crop (Equation (1)), $w_{\mathrm{roi}}$ and $W_{\mathrm{in}}$ are the ROI width and network input width as defined in Equation (3). For the global-search mode (first frame), $x_{\min}=0$ and $w_{\mathrm{roi}}/W_{\mathrm{in}}=W_{0}/W_{\mathrm{in}}$.

By using Gaussian soft labels during training and applying log-domain quadratic peak-refinement decoding during inference, the proposed method reduces the grid quantization error introduced by SimCC discretization without increasing model complexity. Under low-resolution inputs such as 256×256, conventional Argmax decoding only returns the discrete bin center and ignores the intra-bin offset. In contrast, the proposed decoder estimates this local offset by fitting the probability curve around the maximum response, thereby improving localization precision at a low computational cost.

#### 4.3.3. Loss Function

Since the SimCC adopted in this paper reformulates keypoint localization as a classification task, the model outputs probability responses on discretized grids rather than a single coordinate value. To effectively supervise the network in learning the soft labels generated by the SA-SimDR strategy, we select Kullback-Leibler (KL) divergence as the training loss function [30]. Compared with standard cross-entropy, which is commonly used with hard one-hot targets, KL divergence is more suitable for Gaussian soft labels because it measures the discrepancy between complete probability distributions. Therefore, it not only encourages the predicted peak to be close to the ground-truth coordinate, but also constrains the probability decay around the peak. This distribution-level supervision is consistent with the subsequent log-domain peak-refinement decoder, which relies on the local probability morphology near the maximum response.

Specifically, the network outputs predicted probability distributions ${\hat{Q}}_{x}$ and ${\hat{Q}}_{y}$ (obtained after softmax normalization) in the horizontal and vertical directions, which are aligned with the ground-truth Gaussian distribution labels $T_{x}$ and $T_{y}$. The total loss function $L_{total}$ is defined as the weighted sum of the KL divergences for the two independent axes:

(11) $$L_{total}=\frac{1}{N}\sum_{i=1}^{N}\lambda_{i}\left(D_{\mathrm{KL}}\left(T_{x_{i}}∥{\hat{Q}}_{x_{i}}\right)+D_{\mathrm{KL}}\left(T_{y_{i}}∥{\hat{Q}}_{y_{i}}\right)\right)$$

where *N* is the number of keypoints per sample (i.e., N=3 in this work), $\lambda_{i}$ is the visibility weight of the *i*-th keypoint, and the loss is further averaged over the mini-batch during training. For any direction, taking the *x*-axis as an example, the single-item KL divergence is calculated as:

(12) $$D_{\mathrm{KL}}\left(T∥\hat{Q}\right)=\sum_{n}T\left(n\right)\cdot \log\left(\frac{T(n)}{\hat{Q}\left(n\right)}\right)$$

Here, *n* represents the grid index. This function forces the model output to be accurate not only at the peak position but also to possess reasonable confidence decay in distribution morphology by minimizing the asymmetric difference between the predicted distribution and the Gaussian ground truth, thereby providing a high-quality probabilistic basis for subsequent sub-pixel decoding.

## 5. Experiments and Results

To evaluate the proposed lightweight weld seam keypoint detection network under consistent conditions, all experiments were conducted on a workstation equipped with an Intel Xeon Gold 6133 CPU @ 2.50 GHz. The software environment was Ubuntu 20.04.5 LTS with PyTorch 2.0.0. Because industrial sites commonly deploy low-cost and low-power embedded computers, all inference latency results reported in this section were measured under CPU-only settings, with the number of threads fixed to 8 and without GPU acceleration. The training configuration and key hyperparameters are provided in Table 3.

In this section, model size and computational complexity are characterized by the number of parameters and floating-point operations (FLOPs), while localization accuracy is quantified by pixel-distance errors between predicted and ground-truth keypoints. For a visible keypoint instance *i*, let the ground-truth coordinate be $p_{i}=\left(x_{i},y_{i}\right)$ and the prediction be ${\hat{p}}_{i}=\left({\hat{x}}_{i},{\hat{y}}_{i}\right)$ (both in pixels). We define the Euclidean pixel-distance error as

(13) $$e_{i}={∥p_{i}-{\hat{p}}_{i}∥}_{2}=\sqrt{{\left(x_{i}-{\hat{x}}_{i}\right)}^{2}+{\left(y_{i}-{\hat{y}}_{i}\right)}^{2}}.$$

We compute the metrics over all visible keypoint instances in the evaluation set. Let V denote the set of visible instances and let N=|V|. The metrics are computed as

(14) $$\mathrm{MAE}=\frac{1}{N}\sum_{i\in V}e_{i},\;\mathrm{RMSE}=\sqrt{\frac{1}{N}\sum_{i\in V}e_{i}^{2}}.$$

We report errors for the seam center point ($P_{c}$), denoted as SC-MAE/SC-RMSE, and for the two orientation reference points ($P_{l}$ and $P_{r}$), denoted as Ref-MAE/Ref-RMSE.

It should be noted that the above pixel-space errors quantify the visual keypoint localization accuracy in the image domain. They do not have a one-to-one linear correspondence with the final task-space error of robotic welding, because the latter is also affected by camera calibration accuracy, structured-light triangulation error, hand–eye calibration, mechanical execution error, and control latency. Therefore, pixel-level metrics are used in this section to evaluate the perception module itself, while the practical robot-level performance is further assessed through millimeter-level trajectory tracking errors and angular pose errors in the integrated closed-loop experiments.

### 5.1. Detection Performance on Heterogeneous Weld Seams

To select an input resolution suitable for industrial deployment while balancing accuracy against speed, we benchmarked the model’s overall performance across multiple input scales. Table 4 summarizes the comprehensive metrics at 256, 384, and 512.

As the input resolution increases, the localization accuracy improves accordingly: the seam-center-point MAE decreases from 1.89 px to 1.71 px and further to 1.58 px. This improvement, however, is accompanied by additional computational cost, as the FLOPs grow with the input scale and the CPU inference latency rises from 17.01 ms to 21.29 ms and 26.32 ms. Since industrial closed-loop control places a stronger emphasis on update frequency, and the 256×256 setting already provides a center-point MAE of 1.89 px with adequate localization precision for the subsequent closed-loop experiments, 256×256 is adopted as the default resolution for the remaining experiments and system integration.

Using the 256×256 input setting, we then examine the detection performance on the five heterogeneous weld types. The corresponding keypoint localization metrics are reported in Table 5.

Across the five weld categories, the center-point MAE is maintained at 1.89 px, while the reference-point MAE reaches 1.66 px, suggesting reliable generalization under the tested low-resolution setting. The errors are lower for Fillet and Butt-I welds because their groove roots, stripe intersections, and upper-surface endpoints provide relatively clear geometric cues. Lap welds, by comparison, are more affected by strong reflections and by local contour ambiguity around the step. These factors make the semantic boundary between the seam center point and the orientation reference points less distinct, thereby increasing both center-point and reference-point errors. Nevertheless, the center-point MAE of Lap welds remains at a practical level of 2.64 px. This result indicates that the unified three-keypoint formulation is effective across heterogeneous welds, but it also suggests that scenes with locally ambiguous geometry and severe reflection remain more challenging for precise keypoint localization. These results also suggest that combining the unified three-point representation with the proposed network enables a single model to meet fine-grained localization requirements across multiple weld morphologies.

### 5.2. Global Initialization Performance Verification

During the “cold-start” stage, namely the first frame of an automated welding sequence, temporal keypoint priors are not yet available and therefore cannot be used for ROI cropping. This setting is different from subsequent ROI-based tracking frames, where the previous-frame keypoints provide a spatial prior for narrowing the search region. Consequently, the first frame must be initialized by global detection over the input image, and slight differences in accuracy and latency compared with ROI-assisted tracking are expected. Table 6 reports the global initialization performance without ROI assistance.

The results indicate that as the input resolution decreases, the computational cost drops significantly. With 256 × 256 input, FLOPs are 0.24 G and CPU inference latency is about 17 ms, which satisfies the requirements on startup speed and response frequency in industrial settings. Although higher resolutions provide certain accuracy gains (e.g., a center-point MAE of 2.09 px at 512 × 512), latency increases to 27.53 ms, which is more likely to become a real-time bottleneck on low-compute platforms. Since global initialization is only required at the cold-start frame, its role is to provide a reliable initial keypoint state for subsequent ROI tracking rather than to replace the closed-loop tracking process. Considering the trade-off between accuracy and latency, 256 × 256 is a more suitable default setting for industrial startup response in the global-search initialization stage.

### 5.3. Ablation Studies

To verify the effectiveness of the proposed key modules and to clarify their impact on accuracy and speed, ablation studies were conducted under different input resolutions. The baseline is the original SimCC network. We then (i) replace the backbone with the lightweight design, (ii) introduce the log-domain quadratic peak-refinement decoding strategy, and (iii) combine both to form the final model. The results are summarized in Table 7.

After introducing the C3k2-based backbone, at 256×256 resolution the parameter count drops sharply from 67.8 M to 1.4 M, and CPU inference latency decreases from 207.19 ms to 16.82 ms. This demonstrates that the lightweight architecture substantially removes redundant computation while preserving key geometric information, benefiting from an efficient downsampling design and attention enhancement that improve robustness. In addition to reducing computation, the redesigned backbone also improves the coarse feature representation for sparse weld keypoints, as reflected by the lower localization errors compared with the original SimCC baseline at the same resolution.

A direct comparison between “+Backbone” and “Ours” indicates that log-domain quadratic peak-refinement decoding improves keypoint accuracy while introducing virtually no extra runtime overhead. The gain is more evident at lower input resolutions. For the 256×256 input, the center-point MAE decreases from 2.09 px to 1.89 px, with almost no change in inference latency. As the input resolution increases, the effect becomes weaker. At 512×512, the classification bins are already dense enough to reduce much of the quantization error, and the remaining localization error is more likely caused by annotation uncertainty and fluctuations in the laser-stripe halo. This leaves little room for further improvement from coordinate refinement, so the MAE decreases by only 0.04 px at 512×512.

The two modules therefore play complementary roles. The lightweight backbone mainly reduces the parameter count and inference latency while improving coarse feature extraction, whereas the log-domain peak-refinement decoder improves fine localization at a low computational cost by correcting discretization-induced bias. The final “Ours” configuration reflects the combined effect of the lightweight backbone and the proposed decoder. Overall, the 256×256 input with log-domain quadratic peak-refinement decoding provides the best balance between cost and performance, requiring only 0.24 G FLOPs while maintaining practical accuracy and computational efficiency.

### 5.4. Comparative Experiments

For reproducibility and consistent interpretation, we grouped the compared methods into four representative categories. Traditional digital image processing (DIPT) [8,31] was implemented following established pipelines, adapted to our platform, and mainly used for Butt-V weld detection. The distribution-based regression method RLE [32] represents the coordinate-regression paradigm with a relatively high computational cost. YOLOv11 [27] and YOLOv26 [33] were included as object detection methods based on box regression and adapted to weld scenes. The original SimCC was used as the coordinate-classification baseline without the proposed lightweight redesign. Together, these baselines cover the main solution paradigms for weld localization, including traditional image processing, coordinate regression, object detection, and coordinate classification, which enables a representative comparison across different modeling families.

For the YOLO-based annotation scheme, the three keypoints are converted into bounding-box labels. Specifically, a fixed-size 64×64 pixel box is generated around each keypoint, reformulating keypoint localization into a three-class object detection problem. The center of each predicted box is then used as the corresponding keypoint coordinate. The overall comparison results are reported in Table 8.

**Figure 7:**
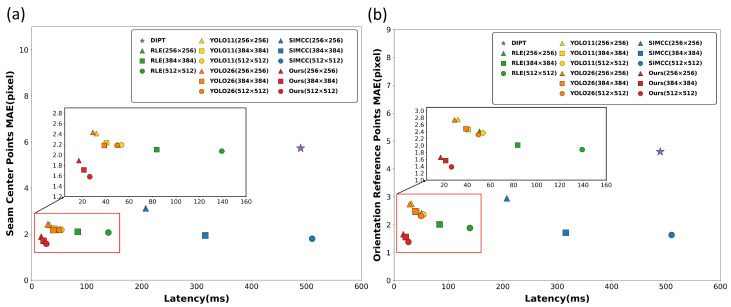
Latency–MAE trade-off across methods and input sizes: (**a**) seam center points; (**b**) orientation reference points.

**Figure 8:**
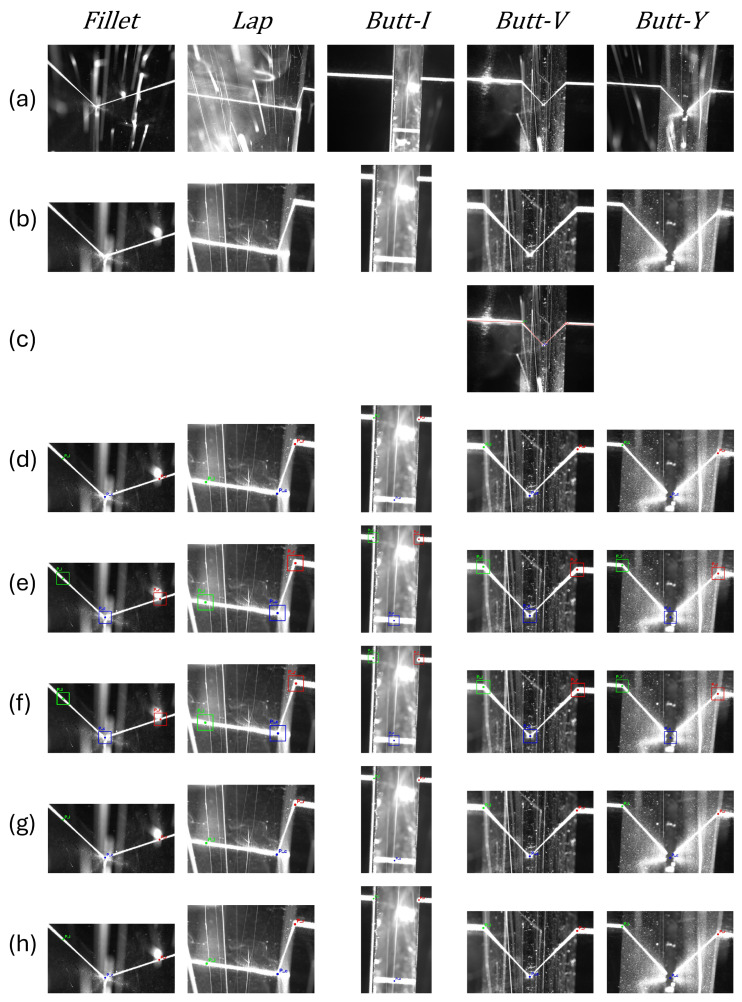
Qualitative comparison across weld types and methods. Columns: Fillet, Lap, Butt-I, Butt-V, Butt-Y. Rows: (**a**) original image; (**b**) ROI; (**c**) DIPT; (**d**) RLE; (**e**) YOLOv11; (**f**) YOLOv26; (**g**) SimCC; (**h**) Ours.

Overall, DIPT shows limited robustness to strong noise and complex reflections, with the largest error and the longest runtime, making it unsuitable for real-time closed-loop control. Compared with YOLOv11 and YOLOv26, the detection frameworks are more versatile, but their box-regression-based localization is less stable for weld seams that are slender and exhibit strong local shape variations. RLE provides reasonable accuracy, yet its much heavier FLOPs burden increases the deployment cost on low-compute devices. Considering accuracy, latency, and computational cost together, the proposed method achieves the most favorable balance at 256 × 256 among the tested settings, indicating practical potential for deployment on low-cost industrial computing platforms. The latency–accuracy trade-off across all methods and input sizes is visualized in Figure 7. Qualitative detection results on five weld types are provided in Figure 8.

### 5.5. System Integration and Experimental Verification

To validate the effectiveness of the proposed detection network based on the unified three-keypoint formulation in real industrial applications, we built a robotic welding experimental platform and conducted trajectory tracking and pose adjustment experiments. The pixel-level errors reported in the preceding subsections characterize the visual localization accuracy of the perception module, whereas the millimeter-level tracking errors and pose angular errors in this subsection characterize the task-space control accuracy of the robotic welding system. This subsection focuses on how the perception output is converted into robot motion commands through a unified perception-to-control mapping, and on the overall closed-loop accuracy of the system.

In the integrated system, each visual feedback cycle includes image acquisition, ROI-based keypoint inference, coordinate transformation from the image frame to the robot base frame, controller communication, and robot motion update. At the default input size, the proposed model achieves a CPU inference latency of 17.01 ms, corresponding to a visual inference rate of more than 50 Hz. Given that the actual welding speed in the tracking experiments was set to 2.5 mm/s, this inference speed is compatible with the closed-loop tracking requirement of the present robotic welding system.

**Figure 9:**
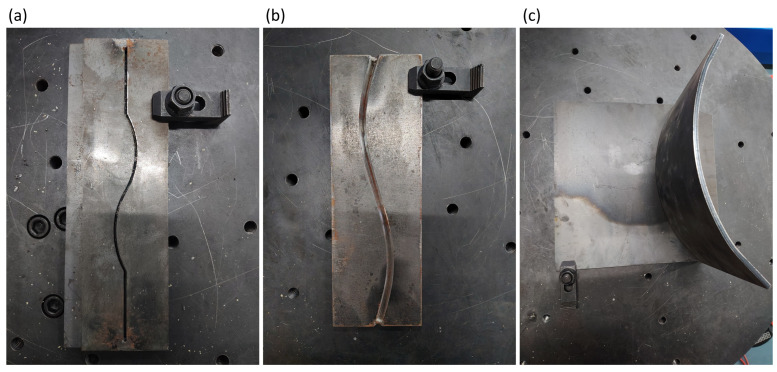
Curved weld workpieces used for closed-loop experiments (before welding): (**a**) Butt-I; (**b**) Butt-V; (**c**) Fillet.

Three typical industrial weld workpieces were selected: Butt-V weld, Butt-I weld, and Fillet weld. The selected workpieces are shown in Figure 9. The robot teaching speed was set to 6 mm/s, and the actual welding speed was set to 2.5 mm/s. The system adopts a geometry-based keypoint-to-command mapping to convert the three predicted keypoints into robot control signals [15], as follows:

- **Trajectory Control (Position):** The seam center point ($P_{c}$) is used as the trajectory reference. The robot TCP is guided to follow the 3D point associated with $P_{c}$, so that the welding torch remains aligned with the seam center.
- **Orientation Control (Pose):** The left orientation reference point ($P_{l}$), seam center point ($P_{c}$), and right orientation reference point ($P_{r}$) jointly provide local geometric cues. The vectors $\vec{P_{c}P_{l}}$ and $\vec{P_{c}P_{r}}$ define the cross-sectional normal plane, from which the target torch attitude, namely roll, pitch, and yaw, is computed to maintain perpendicularity or conform to preset process angles.

**Figure 10:**
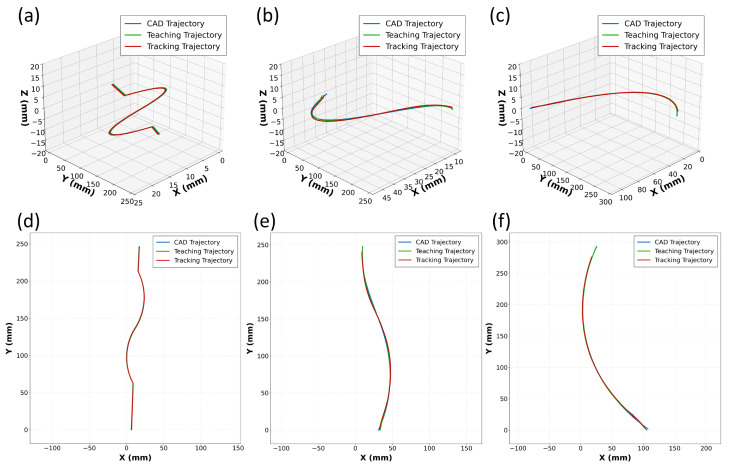
Trajectory comparison among CAD reference, teaching, and vision-based tracking: (**a**) Butt-I weld (3D); (**b**) Butt-V weld (3D); (**c**) Fillet weld (3D); (**d**) Butt-I weld (xy projection); (**e**) Butt-V weld (xy projection); (**f**) Fillet weld (xy projection).

To comprehensively evaluate tracking accuracy, tests were performed on three planar curved weld plates. The CAD theoretical trajectory (blue), teaching trajectory (green), and tracking trajectory (red) were compared. The CAD, teaching, and tracking trajectories are compared in Figure 10. The tracking trajectory closely matches the theoretical trajectory, indicating strong path-following capability.

**Figure 11:**
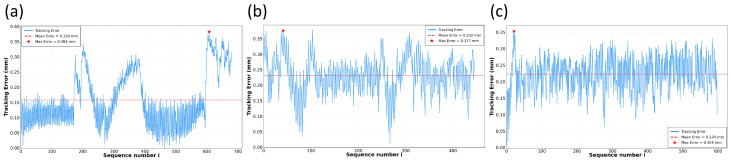
Per-frame trajectory tracking error: (**a**) Butt-I weld; (**b**) Butt-V weld; (**c**) Fillet weld.

For quantitative analysis, the relationship between sequence index and tracking error was recorded. The per-sequence tracking errors are plotted in Figure 11. The statistics show that the average tracking error for the Butt-I weld is 0.159 mm with a maximum error of 0.384 mm; for the Butt-V weld, the average error is 0.232 mm with a maximum error of 0.377 mm; and for the Fillet weld, the average error is 0.224 mm with a maximum error of 0.354 mm. All tracking errors remain in the sub-millimeter range, which satisfies engineering tolerances for weld-path accuracy and, in turn, supports the use of $P_{c}$ as a reliable position-feedback signal.

The temporal behavior of the tracking errors also provides an initial indication of tracking stability. In the proposed system, the keypoints are re-estimated from the current image at every visual feedback cycle, rather than being obtained by integrating historical motion as in odometry- or SLAM-like estimation. The previous-frame prediction is only used to define the ROI search region and is not directly accumulated as the current coordinate output. Therefore, the system is less prone to monotonic drift caused by coordinate integration. As shown in Figure 11, the per-frame errors fluctuate within a bounded range and do not exhibit an obvious monotonically divergent trend in the tested sequences. Nevertheless, long-duration drift benchmarking over longer welding sequences and more diverse disturbances has not yet been systematically conducted, which remains a limitation of the present evaluation.

#### Actual Welding Results

**Figure 12:**
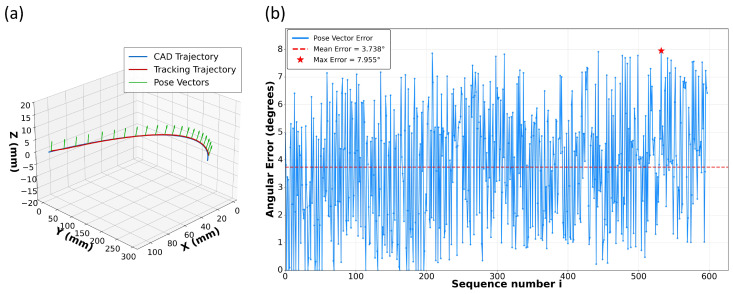
Pose adaptation results on a fillet weld: (**a**) 3D tracking trajectory with estimated pose vectors; (**b**) per-frame angular error.

Additional pose-adaptation tests were carried out on fillet-weld workpieces. Because manual teaching cannot ensure that the torch pose stays perfectly aligned with the theoretical normal direction over the entire process, we primarily examine the deviation between the designed nominal pose and the pose executed in practice. As the seam proceeds along the arc, the estimated pose vector varies smoothly and continuously, while maintaining good consistency with the weld normal plane. The pose vectors and the corresponding angular errors along the trajectory are presented in Figure 12. The mean angular error is 3.738°, and the maximum error is 7.955°. This accuracy is sufficient for arc-welding operations, as it helps stabilize the molten pool and supports consistent bead formation. It also indicates that the proposed three-keypoint formulation provides useful pose cues for robotic control.

**Figure 13:**
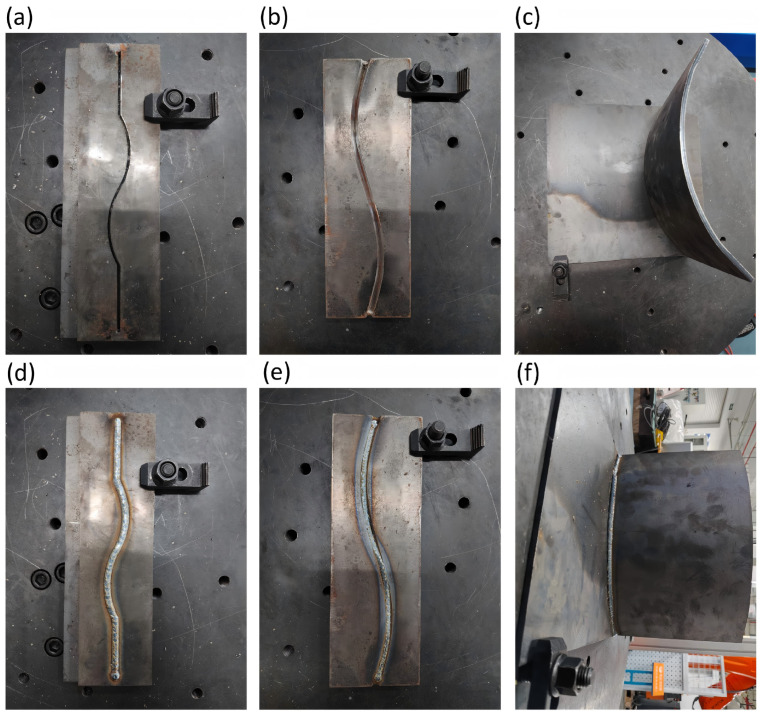
Macroscopic appearance of workpieces for welding validation: (**a**) Butt-I weld (pre-weld); (**b**) Butt-V weld (pre-weld); (**c**) Fillet weld (pre-weld); (**d**) Butt-I weld (post-weld); (**e**) Butt-V weld (post-weld); (**f**) Fillet weld (post-weld).

To further evaluate the overall control performance, Gas Metal Arc Welding (GMAW) trials were conducted after the tracking experiments. The appearances of the three workpieces before and after welding are shown in Figure 13. At the macroscopic level, the welded seams show continuous and uniform ripples, with no evident defects such as undercutting or seam deviation. These results indicate that the proposed framework, which integrates deep-learning-based perception with control, can maintain stable welding quality under strong visual disturbances.

## 6. Conclusions

This paper presents an end-to-end lightweight framework for weld seam keypoint detection and robotic tracking in industrial welding scenarios affected by arc glare, spatter, smoke, surface reflection, and heterogeneous seam geometries. By formulating weld perception as a sparse three-keypoint localization task, the proposed framework produces a compact perception output that can be directly used for trajectory correction and torch-pose estimation.

The proposed method has three main characteristics. First, a unified control-oriented three-keypoint formulation is introduced, in which one seam center point and two orientation reference points describe different weld geometries with consistent semantics and decouple position control from orientation estimation. Second, a lightweight SimCC-based pipeline is developed by replacing the heavy original backbone with a compact C3k2-based feature extractor, thereby reducing computational cost while preserving weld-related geometric cues. Third, a non-parametric log-domain quadratic peak-refinement decoder refines continuous coordinates from SimCC classification distributions and mitigates discretization-induced coordinate bias without adding trainable parameters. Combined with temporally guided ROI tracking, these designs support efficient closed-loop weld tracking.

Experiments show that, with an input size of 256×256, the proposed model contains only 1.4 M parameters, requires 0.24 G FLOPs, and achieves a CPU inference latency of 17.01 ms. On the validation set covering five representative weld types, it obtains a seam-center-point MAE of 1.89 px and a reference-point MAE of 1.66 px. In the integrated robotic welding experiments, the framework achieves sub-millimeter trajectory tracking accuracy, with the lowest mean tracking error of 0.159 mm obtained on the Butt-I weld, and obtains an average orientation error of 3.738° in the fillet-weld pose-adaptation experiment.

Several limitations remain. The current evaluation is mainly based on a self-built dataset and a specific structured-light robotic welding platform, and cross-platform generalization under different cameras, laser configurations, welding parameters, and workpiece materials still requires further validation. The temporally guided ROI strategy may also fail under severe occlusion, abrupt seam-position changes, abnormal robot motion, or strong transient disturbances, while long-sequence drift and failure recovery have not yet been systematically quantified. In addition, the preset distance e=300 pixels for Fillet and Lap welds is tied to the current imaging scale and may need re-tuning for different sensors or weld sizes. Future work will focus on external validation, robust ROI failure recovery, automatic re-initialization for long-duration tracking, and adaptive keypoint definitions for non-standard or severely deformed weld seams.

## Figures and Tables

**Table 1:** Detailed hardware configuration of the experimental platform.

Equipment	Model/Specification
Robot	SIASUN SR12A-12/1.46
Vision Sensor	Custom-developed structured-light sensor
Robot Controller	SIASUN SRC E5
Welding Machine	AOTAI NBC-500RP Plus
Welding Feeder	AOTAI CS-501-500
Shielding Gas	CO_2_

**Table 2:** Distribution of weld seam images in the dataset.

Dataset Split	Fillet	Lap	Butt-I	Butt-V	Butt-Y	Total
Training Set	1039	1001	1140	1172	1012	5364
Validation Set	251	251	274	282	249	1307
Total	1290	1252	1414	1454	1261	6671

**Table 3:** Training settings and hyperparameters for the proposed network.

Hyperparameter	Value
Optimizer	Adam
Base learning rate	$5\times 10^{-4}$
Weight decay	$1\times 10^{-5}$
Batch size	32
Training epochs	400
LR schedule	Multi-Step
LR decay steps	[350, 380]
Sigma (σ)	4.0
Split factor (*k*)	2
Input size	256×256

**Table 4:** Performance comparison at different input resolutions (ROI input).

Input Size	Params (M)	Latency (ms)	FLOPs (G)	SC-MAE (px)	SC-RMSE (px)	Ref-MAE (px)	Ref-RMSE (px)
256×256	1.4	17.01	0.24	1.89	2.44	1.66	2.25
384×384	1.6	21.29	0.55	1.71	2.32	1.48	2.06
512×512	1.9	26.32	0.98	1.58	2.21	1.38	1.95

**Table 5:** Detailed detection metrics across different weld types (Input: 256×256).

Metric	Fillet	Lap	Butt-I	Butt-V	Butt-Y	Average
**SC-MAE** (px)	1.48	2.64	1.69	1.71	1.99	1.89
**SC-RMSE** (px)	1.65	3.26	2.27	2.42	2.39	2.44
**Ref-MAE** (px)	1.32	2.48	1.43	1.66	1.47	1.66
**Ref-RMSE** (px)	1.61	3.10	2.07	2.34	1.88	2.25

**Table 6:** Global initialization performance without ROI assistance at different input resolutions.

Input Size	Params (M)	Latency (ms)	FLOPs (G)	SC-MAE (px)	SC-RMSE (px)	Ref-MAE (px)	Ref-RMSE (px)
256×256	1.4	17.47	0.24	2.38	3.21	2.75	3.78
384×384	1.6	22.19	0.55	2.14	2.81	2.52	3.60
512×512	1.9	27.53	0.98	2.09	2.78	2.50	3.54

**Table 7:** Ablation study results of different improvement modules (ROI input).

Method	Input Size	Params (M)	Latency (ms)	FLOPs (G)	SC-MAE (px)	SC-RMSE (px)	Ref-MAE (px)	Ref-RMSE (px)
Baseline	256×256	67.8	207.19	21.00	3.12	3.75	2.95	3.66
	384×384	77.8	315.54	47.27	1.94	2.50	1.71	2.13
	512×512	97.2	510.18	84.06	1.80	2.48	1.63	2.01
+Backbone	256×256	1.4	16.82	0.24	2.09	2.68	1.76	2.44
	384×384	1.6	21.18	0.55	1.72	2.32	1.56	2.09
	512×512	1.9	26.50	0.98	1.62	2.25	1.40	1.98
+Log-Domain Peak-Refinement	256×256	67.8	218.15	21.00	2.10	2.62	1.84	2.28
	384×384	77.8	320.59	47.27	1.92	2.49	1.55	2.09
	512×512	97.2	511.06	84.06	1.72	2.33	1.55	1.96
**Ours**	256×256	**1.4**	**17.01**	**0.24**	**1.89**	**2.44**	**1.66**	**2.25**
	384×384	**1.6**	**21.29**	**0.55**	**1.71**	**2.32**	**1.48**	**2.06**
	512×512	**1.9**	**26.32**	**0.98**	**1.58**	**2.21**	**1.38**	**1.95**

Note: Bold values indicate the best performance for the same input size.

**Table 8:** Comparison with representative methods under different input resolutions (ROI input).

Method	Input Size	Params (M)	Latency (ms)	FLOPs (G)	SC-MAE (px)	SC-RMSE (px)	Ref-MAE (px)	Ref-RMSE (px)
DIPT	—	—	489.26	—	5.73	8.46	4.62	5.85
RLE	256×256	23.58	50.50	5.36	2.19	3.25	2.41	3.00
	384×384	23.58	83.53	12.08	2.10	2.99	2.01	2.73
	512×512	23.58	139.22	21.47	2.07	2.86	1.88	2.62
YOLOv11	256×256	2.590	31.97	0.515	2.41	3.15	2.75	3.64
	384×384	2.590	40.60	1.160	2.23	3.15	2.46	3.51
	512×512	2.590	53.49	2.062	2.19	3.01	2.36	3.23
YOLOv26	256×256	2.505	29.04	0.462	2.43	3.23	2.74	3.59
	384×384	2.505	38.75	1.040	2.18	2.96	2.48	3.60
	512×512	2.505	49.60	1.848	2.18	2.95	2.31	3.11
SimCC	256×256	67.8	207.19	21.00	3.12	3.75	2.95	3.66
	384×384	77.8	315.54	47.27	1.94	2.50	1.71	2.13
	512×512	97.2	510.18	84.06	1.80	2.48	1.63	2.01
**Ours**	256×256	**1.4**	**17.01**	**0.24**	**1.89**	**2.44**	**1.66**	**2.25**
	384×384	**1.6**	**21.29**	**0.55**	**1.71**	**2.32**	**1.48**	**2.06**
	512×512	**1.9**	**26.32**	**0.98**	**1.58**	**2.21**	**1.38**	**1.95**

Note: Bold values indicate the best performance for the same input size.

## Data Availability

The data presented in this study are available on request from the corresponding author due to privacy and ethical restrictions.
